# Effects of Pulsed Current on the Microstructure and Properties of Laser Cladded TC17 Titanium Alloy

**DOI:** 10.3390/ma17010091

**Published:** 2023-12-23

**Authors:** Zhao Liu, Ping Liu, Liucheng Zhou, Lingfeng Wang

**Affiliations:** 1 School of Aeronautics, Chongqing Jiaotong University, Chongqing 400074, China; liuzhao528@163.com; 2 Science and Technology on Plasma Dynamics Laboratory, Air Force Engineering University, Xi’an 710038, China; happyzlch@163.com (L.Z.); lf_wang_afeu@163.com (L.W.)

**Keywords:** laser cladding, pulsed current, titanium alloy, microstructure, preferential growth, corrosion

## Abstract

In this study, a titanium alloy substrate was cladded with TC17 titanium alloy powder using the pulsed-current (PC)-assisted laser cladding technique. The primary objective of this research was to assess the impact of varying pulsed current intensities on the morphology, microstructure, and properties of samples. It is observed that the utilization of pulsed currents significantly enhances the metallurgical adhesion between the samples, concurrently diminishing the occurrence of porosity within the cladding layer. The incorporation of a pulsed current also has a positive impact on the microhardness and corrosion resistance of the samples. Furthermore, the synergistic influence of laser energy and a pulsed electrical current is found to promote a structural evolution in materials towards a state with lower electrical resistance. The introduction of a pulsed current leads to preferential growth of β grains with <100>// cladding direction in the cladding zone and obtains the typical {100} < 001 > cube texture, while the substrate zone exhibits a distinctive stripe-like configuration formed by the primary α-phase constituents. The outcomes of this study show the pivotal role of pulsed currents as an auxiliary technique for enhancing the properties and effecting microstructural modifications in titanium alloys during the laser cladding process.

## 1. Introduction

Currently, a significant proportion of titanium alloy components are fabricated through traditional forging techniques, a method known to exhibit suboptimal material utilization rates. In contrast, additive manufacturing (AM) technology demonstrates pronounced advantages, particularly in terms of complex manufacturing and net shape forming [1,2,3]. Laser cladding is a type of additive manufacturing utilizing a laser beam to melt material powders and fuse them to the surface layer of the substrate material [4,5]. Such a method is predominantly employed for the remediation of defects and the application of reinforced coatings in titanium alloy components, contributing positively to both their longevity and their enhanced properties.

However, the intrinsic rapid rates of heating and rapid rates of cooling in the laser cladding process often lead to an organizational mismatch between the cladding and base material zones and the development of defects (such as a lack of fusion and porosity) within the cladding zone [6,7]. These issues adversely affect the mechanical properties and metallurgical bonding ability of titanium alloy materials, thus significantly impeding the further development and application of laser cladding technology.

In order to mitigate the adverse effects of the laser cladding process on the production and repair of titanium alloy components, previous studies have endeavored to improve the properties of the titanium alloy cladding layer and minimize internal defects through the adjustment of laser cladding process parameters, the implementation of heat treatments, and other techniques [8,9,10]. However, the widespread adoption of these methods is impeded by the limited range of available materials and the extended exposure to high-temperature conditions. In this context, the auxiliary field, a technique not directly interfacing with the material, can be employed to change the microstructure and enhance the mechanical properties of the material during and after the cladding process. Thus, it has widespread applications in the manufacturing fields of casting, welding, and laser processing. The auxiliary field encompasses modalities such as mechanical vibration [11], the ultrasonic field [12], and the electromagnetic field [13], among others. Among these methods, mechanical vibration and the ultrasonic field exhibit significant energy attenuation, so a substantial portion of the energy fails to reach the molten pool, resulting in a limited impact on the molten pool’s bottom. In the case of electromagnetic field assistance, achieving a uniform distribution of the electromagnetic field over the surface of large parts poses challenges. Additionally, the electromagnetic field apparatus is relatively intricate, and its deployment is constrained by spatial limitations when inert gas cladding conditions are required.

Pulsed currents are often used in areas of material processing and surface modification due to its advantages of high energy density, precise control, and diverse applicable materials. As investigation progressed, researchers have uncovered that pulsed current applied to metallic materials generates both thermal and non-thermal effects, mainly including the Joule heating effect [14], skin effect [15], magnetic compression effect [16], electron migration [17], electron wind force [18], etc. These effects of pulsed currents have a positive impact on reducing internal defects in components, improving refinement of grains, and improving mechanical properties. Xie et al. [19] combined pulsed currents with the laser cladding process to investigate the effect of pulsed currents on the microstructure and porosity of a laser-cladded Cobalt-based FGH95 alloy. Studies have shown that the magneto constriction effect produced by pulsed currents decreased the porosity of the cladding layer, and the added supercooling degree refined the grain of the cladding layer. Xie et al. [20] investigated the effect of EST on the organization and mechanical properties of thin-walled specimens of a β Ti-55531 alloy and found that the ultra-high current causes the Joule heating effect and the electron wind force, which speeds up the atomic diffusion rate and triggers local phase transitions on the needle-like α tips. This leads to the spheroidization phenomenon of the α precipitate phase. The content of α-phase increases slightly, the grain size of β is refined, and the strength and toughness of β Ti-55531 are improved after EST. At the same time, the pulsed current also has a certain effect on the structural evolution of materials. Rahnama et al. [21] investigated the effect of electropulsing on niobium carbide at an elevated temperature. Electropulsing treatment has resulted in an increase in nucleation rate, and therefore in production of finer grain sizes. Additionally, it has been discovered that a semi-transformed pearlite structure develops in a manner where the grains align in a direction parallel to the flow of the electric current. Chao Wu et al. [22] studied the effect of electropulsing treatment on the texture-changed law and mechanism of 35CrMo steel. This work found that electropulsing treatment can inhibit the growth of grains with the original texture at a relatively lower current density, and at the grain growth stage, promote the growth of grains with <100>//CD via a higher current density.

At present, nevertheless, there are few systematic studies on pulsed-current-assisted laser cladding. The impact of pulsed currents on the cladding zone during laser cladding of titanium alloys was mainly emphasized in previous studies. However, the combined effects of laser energy and pulsed currents on the substrate zone are often overlooked, resulting in an incomplete evaluation of the influence of pulsed currents on the laser repair of titanium alloys. In this article, the TC17 material was used as the substrate, and the samples were obtained through pulsed-current-assisted laser cladding. The effects of pulsed current intensity on the cross-sectional morphology, porosity, grain orientation, and microstructure of the fused cross-section region of TC17 were investigated, and the mechanism of its action was discussed. The effects of pulsed currents on the microhardness and corrosion properties of TC17 samples were also tested and analyzed. The results of the study have a positive effect on improving the comprehensive performance of titanium alloy cladding materials and provide a reference for the research of laser cladding assisted by pulsed currents.

## 2. Materials and Methods

### 2.1. Materials

In this experiment, a TC17 titanium alloy was employed as the substrate material, possessing dimensions of 128 mm × 24 mm × 10 mm. The material chosen for laser cladding was a TC17 spherical powder. The composition of powder is presented in Table 1, and its particle size is observed to range between 60–145 μm.

### 2.2. PC-Assisted Laser Cladding Experiment

**Figure 1:**
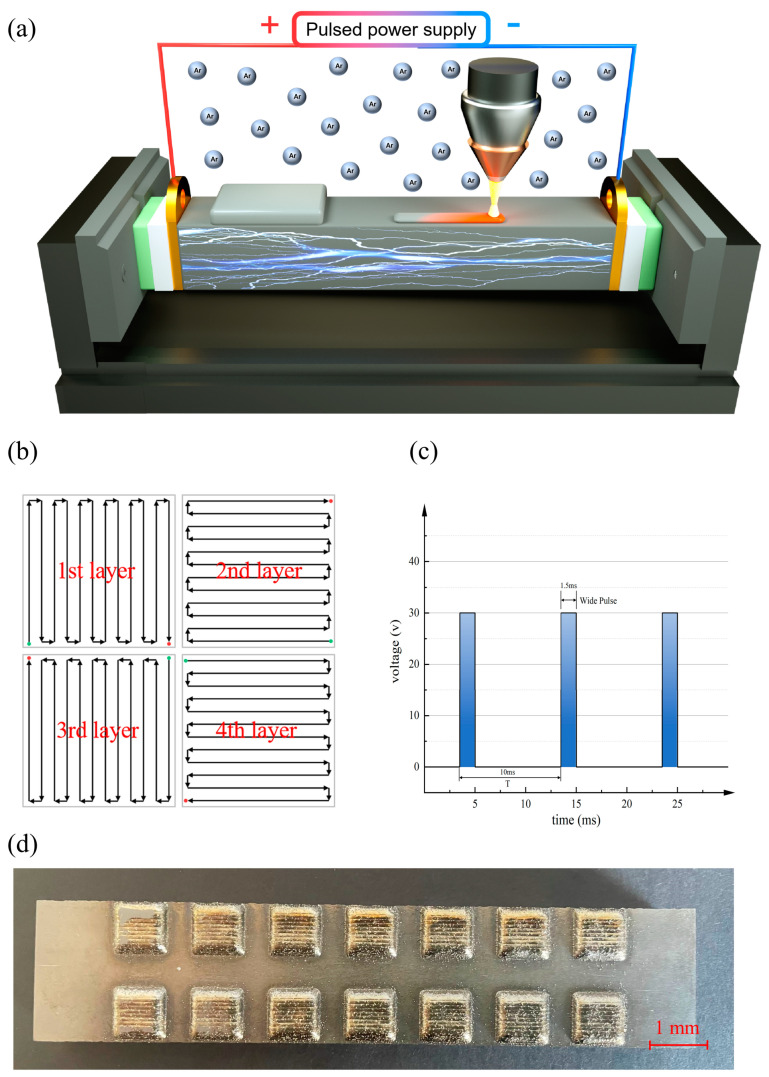
(**a**) Schematic diagram of the experiment; (**b**) Scanning strategies of the laser cladding process; (**c**) The waveform diagram of pulsed current; (**d**) The laser-cladded samples.

The laser cladding experiment was conducted utilizing equipment (Xi’an, China, Xi’an Air & Space Electromechanical Intelligent Manufacturing Co.). A laser power of 1.5 kW was employed, accompanied by a scanning rate of 3 mm/s, a beam diameter of 0.8 mm, and a powder feed rate set at 2.5 g/min. The entire laser cladding procedure was undertaken in the presence of an argon gas flow, maintaining an oxygen content below 60 ppm. During the cladding procedure, the pulsed current was introduced. The TC17 substrate is positioned between two copper electrodes, which are in turn isolated from the fixture by alumina ceramic and FR4 water glass to ensure the passage of pulsed current through the substrate, as shown in Figure 1a. The scanning pattern of the laser cladding process is shown in Figure 1b, and the scanning path is carried out using the layer-by-layer cross, with the thickness of a single layer of the cladded samples being about 1 mm. This approach results in a relatively flat surface and reduces the porosity inside the samples due to the morphology of the molten pool. The ultimate dimensions of the cladded sample stand at 8.8 mm × 8.8 mm × 2.7 mm, as shown in Figure 1d. Figure 1c shows the power supply’s output pulsed current waveform, with a voltage of 30 V, a frequency of 100 Hz, and a duty cycle of 15%. To investigate the influence of pulsed current magnitude on the experiment, the experimental procedure is segmented into four distinct groups, as shown in Table 2.

### 2.3. Performance Testing and Microstructural Characterization

First, the samples with different parameters were cut along the radial direction, then the cross-sectional area of the samples was ground with SiC paper from 240 grit to 5000 grit, polished with diamond powder to a mirror finish, and finally, the samples were ultrasonically cleaned in an ethanol solution. The three primary areas of the sample cross-section were identified as the laser-cladded zone (LCZ), the heat-affected zone (HAZ), and the substrate zone (SZ).

Phase identification of LCZ and HAZ was inspected using XRD equipment (PANalytical Empyrean Series 2, PANalytical B.V., Almelo, The Netherlands) with a Cu-Kα radiation source. The data was collected over 2θ in the range of 30 to 80°with a scanning velocity of 5°/min. Then, the cross-section areas of the samples were etched with Keller’s reagent ($HF:{HNO}_{3}:H_{2}O=1:2:5$) and examined using a scanning electron microscope (EVO10, Carl Zeiss AG, Jena, Germany) to observe the morphology and pore distribution. The images of the morphology and pore were analyzed using ImageJ software (ImageJ 2.9.0) to obtain parameters of section zone size, and statistics of the porosity and pore quantity.

The microscopic morphology of the cross-section area at different gradients was observed through optical microscopy (ZEISS-Axiocam208, Carl Zeiss AG, Jena, Germany) and a scanning electron microscope. Electrolytic polishing was performed on the samples to mitigate surface mechanical stresses prior to EBSD analysis. Grain orientation and textural characterization were analyzed by the EBSD attachment integrated with the scanning electron microscope (ZEISS-Gemini300, Carl Zeiss AG, Jena, Germany). Processing and analysis of the EBSD data were accomplished using Channel 5 software (Oxford Instruments, Abingdon, UK), facilitating a detailed comparison of grain orientation, texture, and grain boundary distribution conditions pre- and post-pulsed current assistance.

The microhardness was measured using a microhardness tester (HXD-1000TMC/LCD, Sunny HengPing Instrument, Shanghai, China) from the top center downward along the vertical direction. A test point was taken every 0.5 mm, the testing load was 200 N, and the dwelling time was 30 s. To guarantee the precision of the hardness data, three or more measurements were taken around the same point to obtain the mean of the hardness distribution curves.

To investigate corrosion resistance, a standard three-electrode workstation (CHI 660E, Chenhua Instrument Co., Shanghai, China) was used in a 3.5 wt% NaCl electrolyte with a pH of 1.0, adjusted with hydrochloric acid. The reference electrode (RE) chosen was Ag/AgCl, while the auxiliary electrode was a platinum gauze. The working electrode (WE) was the sample itself. Potentiodynamic polarization (PP) measurements were conducted, ranging from −1.0 V to +2.0 V vs. Ag/AgCl, with a scanning rate of ${1\;mV\cdot s}^{-1}$. The outcome of the potentiodynamic polarization was analyzed using the extrapolation method.

## 3. Results

### 3.1. Macromorphology of the Cladding Sample

**Figure 2:**
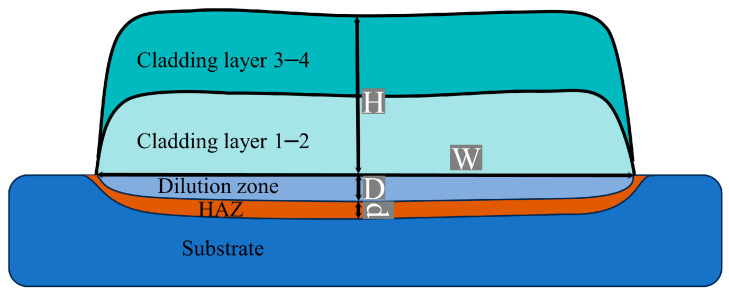
Schematic diagram of cladding sample cross section.

**Figure 3:**
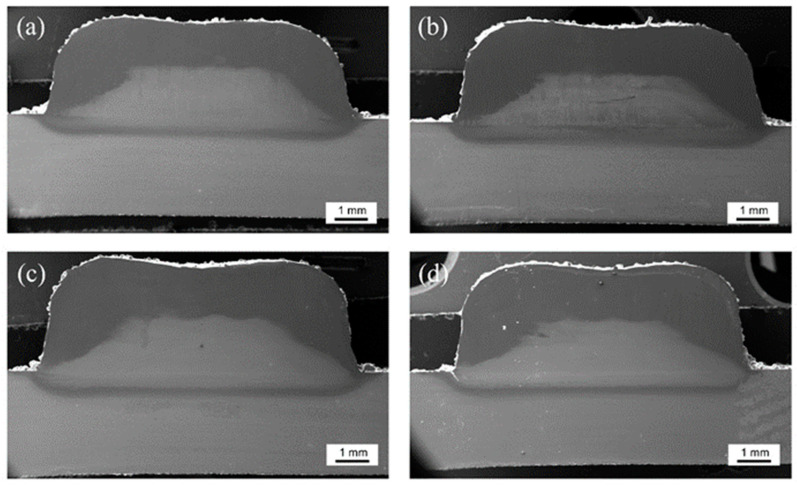
Macromorphologies of the cross section of the cladding samples (**a**) NO-PC; (**b**) PC-10; (**c**) PC-30; (**d**) PC-50.

The cross-sectional macroscopic morphology model of the sample is shown in Figure 2. The cross-section of the sample consisted mainly of the laser-cladded zone (LCZ), the heat-affected zone (HAZ), and the substrate zone (SZ). The cross-sectional morphology of the samples under different pulsed current intensities taken by an optical microscope (OM) is shown in Figure 3. The surface outlines of the cladding zone of the various samples were wavy. The metallurgical bond between the cladding zone and the substrate zone was excellent.

#### 3.1.1. Dilution Rate

The dilution rate refers to the degree of change in the composition of the cladding alloy caused by the melting of the substrate during the laser cladding process. The parameters of the geometry of the cladded samples are shown in Figure 2, and according to the specified parameters shown in the figure, the dilution is:

(1) $$\eta =\frac{D\times W}{(H+D)\times W}\times 100\%$$

where η is the dilution of the cladded sample, H is the average thickness of the dilution zone, D is the average height of the cladding layers, W is the average widths of the cladding layers, and d is the average widths of the heat-affected zone.

The parameters related to the geometric dimensions of the sample sections at different pulsed currents are shown in Table 3. The dilution rate of the samples are positively correlated with the pulsed current. The dilution rate of samples was increased by the larger pulsed current. The dilution rates for samples NO-PC, PC-10, PC-30, and PC-50 were 18.87%, 19.01%, 19.22%, and 19.46%, respectively. Concurrently, the HAZ width of the PC-50 sample was reduced in comparison with the NO-PC sample. Elevated dilution rates and reduced HAZ widths, influenced by the pulsed current, increased the level of metallurgical bonding and diminished the likelihood of defects.

The dilution rate depends mainly on the absorption of laser energy by the cladding surface. According to the Fresnel equation, the laser absorptivity of the metal can be expressed as:

(2) $$A=2\sqrt{\frac{2c\varepsilon_{0}}{\lambda \sigma }}$$

where A is laser absorptivity of the metal, σ is free electron density on the surface of the metal, c is the speed of light, λ is the wavelength of laser radiation, and $\varepsilon_{0}$ is the vacuum permittivity. The speed of light, the wavelength of laser radiation, and vacuum permittivity do not change when the laser power is determined. Thus, the predominant factor that influences the laser absorptivity in the metallic sample is the free electron density. Upon the metallic surface resides a substantial population of free electrons. When the metallic surface is subjected to laser irradiation, most of the laser energy is absorbed by free electrons [23]. Introducing a pulsed current tends to diminish the density of the free electrons on the metallic surface, allowing a portion of the laser to traverse the free electron barrier, eventually being absorbed in the form of energy by the bound electrons within the metal material. The enhancement in laser absorption causes a heat gain effect, promoting the melting of the substrate zone and increasing the dilution rate.

#### 3.1.2. Pore State

**Figure 4:**
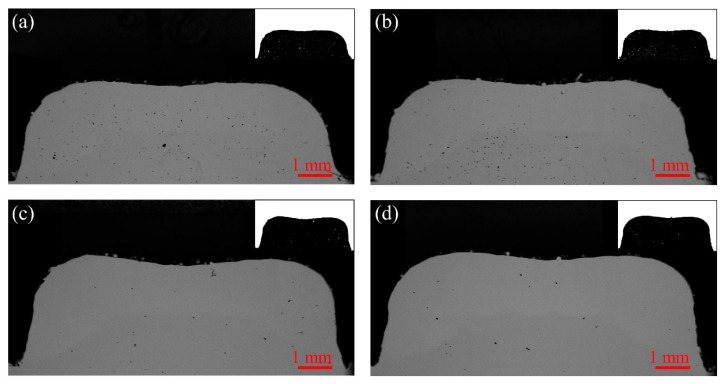
The morphology of the LCZ cross sections (**a**) NO-PC; (**b**) PC-10; (**c**) PC-30; (**d**) PC-50.

**Figure 5:**
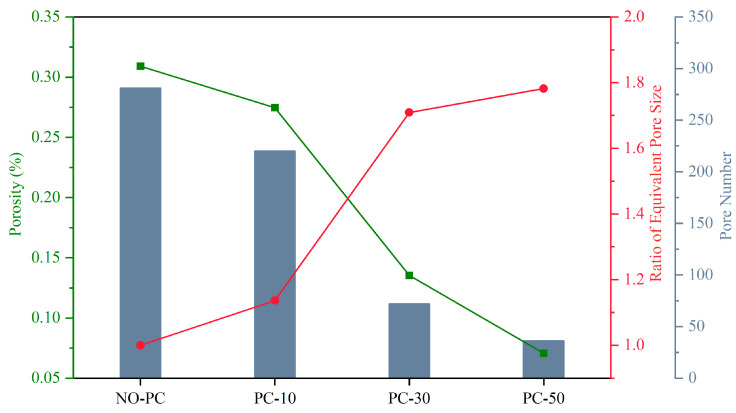
Porosity, ratio of equivalent pore size, and pore number of the NO-PC sample, the PC-10 sample, the PC-30 sample, and the PC-50 sample.

The cross-sectional morphology of the laser cladding zone for different samples is shown in Figure 4a–d. Figure 4a represents the pore state of the LCZ in the absence of a pulsed current. The quantity of pores was considerable and regularly dispersed. Conversely, Figure 4b–d represents the pore state of the LCZ in the presence of a pulsed current. The quantity of pores decreased significantly with higher current intensities, especially the number of small pores. The relationship of the pores under different pulsed current parameters is shown in Figure 5. The pore counts of samples NO-PC, PC-10, PC-30, and PC-50 were 281, 220, 69, and 36, respectively. The count of pores in the PC-50 sample was reduced by 87% compared to the NO-PC sample. Porosity refers to the proportion of the total pore area to the area of the cladding zone. Without pulsed current, the porosity of the sample was 0.31%. With the pulsed current assist, it can be seen that the porosity decreases significantly with the increase in current intensity. The porosity reached its lowest at a current of 50 A, which was 0.07%. The porosity of the 50-PC sample decreased by 77% compared to the porosity of the NO-PC sample. The equivalent pore size was determined based on the correlation between pore number and porosity. It represents the relative size of individual pores within the laser cladding zone. Its value can be seen to increase with the assistance of the pulsed current, which means that the pulsed current increases the pore size in the sample. The pulsed-current-induced fusion of the pores leads to this result. As the pulse current increased, the tendency of pore fusion became more obvious. Compared to the equivalent pore size of NO-PC, the equivalent pore sizes of PC-10, PC-30, and PC-50 were increased by 1.14, 1.71, and 1.78 times, respectively.

#### 3.1.3. The Effect of Pulsed Current

**Figure 6:**
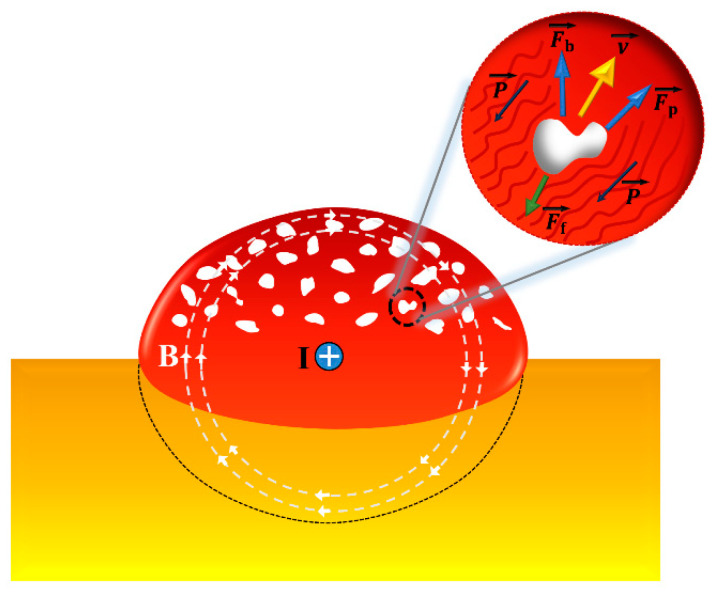
Schematic diagram of the internal motion of the molten pool affected by the pulsed current.

The motion mechanism of the melt pool during the cladding process, assisted by the pulsed current, is illustrated in Figure 6. The current is mostly distributed on the surface of the substrate due to the skin effect [19]. Consequently, the majority of this current is localized within the melt pool during the laser cladding process. The flow of the electric current created a self-induction magnetic field inside and outside the melt pool. Considering the axial symmetry of current distribution and according to Kirchhoff’s current law, the magnetic flux density along the radius can be expressed as [24]:

(3) $$\vec{B}=\frac{\mu r}{2\pi R^{2}}\vec{I}=\frac{\mu r}{2}\vec{J}$$

where μ is the magnetic permeability, I is the current through the melt pool, J is the current density, r is the distance from any point to the axis, and R is the radius of the melt pool.

In the close surroundings of the molten pool, the combined action of the electric current and the induced magnetic field produces the Lorraine magnetic force, which can be expressed as:

(4) $$\vec{P}=\vec{J}\times \vec{B}=-\frac{\mu J^{2}r}{2}\vec{\iota_{1}}=-\frac{\mu I^{2}r}{2\pi^{2}R^{4}}\vec{\iota_{1}}$$

where $\vec{\iota_{1}}$ is the unit vector. The Lorraine magnetic force direction is oriented toward the central axis of the shaft, causing the convergence of liquid metal within the molten pool and the molten substrate toward the shaft’s center. The electromagnetic pressure is greatly correlated with the position of the metallic liquid. The nearer the liquid metal is to the surface of the molten pool, the greater the electromagnetic pressure acting upon it. Additionally, an augmentation in the pulsed current results in a more conspicuous magnetic compression effect. The decrease in the height and the increase in the width of the cladding zone can be attributed to this effect, as shown in Table 3.

With the assistance of a pulsed current, the pores within the melt pool are mainly affected by electromagnetic repulsion, buoyancy, and flow resistance. The resistivity of the gas in the pores is much higher than that of the metal liquid in the melt pool, leading to diminished electromagnetic forces acting on the pores in contrast to the liquid metal. Consequently, the pores are subjected to a relatively equivalent reverse repulsive force. The electromagnetic repulsion exerted on spherical pores within the melt pool can be expressed as [25]:

(5) $$\vec{F_{p}}=-\frac{3}{2}\frac{\sigma_{m}-\sigma_{g}}{2\sigma_{m}+\sigma_{g}}\frac{\pi {d_{p}}^{3}}{6}\vec{P}$$

where $\sigma_{m}\;$is the conductivity of the metal liquid, $\sigma_{g}$ is the conductivity of gas, and $d_{p}$ is the diameter of a spherical pore. The pores are driven towards the edge of the melt pool by electromagnetic repulsion, which opposes the direction of the electromagnetic pressure. Due to the conductivity of gases being much higher than that of molten metals, Equation (5) is subsequently streamlined as follows:

(6) $$\vec{F_{p}}=-\frac{3}{4}\frac{\pi {d_{p}}^{3}}{6}\vec{P}$$

The buoyancy force exerted by the pores in the metallic liquid is calculated as:

(7) $$\vec{F_{b}}=\rho_{m}Vg\vec{\iota_{2}}$$

where $\vec{\iota_{2}}$ is the unit vector, $\rho_{m}$ is the density of the metal liquid in the molten pool, V is the volume of pores, and g is the gravitational acceleration. The direction of $F_{b}$ is vertically upwards along the horizontal plane.

When electromagnetic repulsion and buoyancy induce pore motion, the resultant fluid resistance arising between the pores and the metallic liquid serves as an impediment to their motion. The flow resistance is expressed as follows [26]:

(8) $$\vec{F_{f}}=\frac{1}{6\tau_{P}}\pi d_{p}^{3}\rho_{m}(\vec{u}-\vec{v})$$

where $\tau_{P}$ is the particle response time, $\vec{u}$ is the flow velocity of the molten metal, and $\vec{v}$ is the relative flow velocity of the pore. When the Reynolds number of the relative motion is much less than 1, particle response time could be calculated [27]:

(9) $$\tau_{P}=2\rho_{g}{d_{p}}^{2}/(9\mu )$$

where $\rho_{g}$ is the density of gas, and μ is the dynamic viscosity of metallic fluids. The dynamic viscosity has a great correlation with the melt pool temperature. A high melt pool temperature can accelerate the thermal motion of molecules and weaken intermolecular interactions, thereby reducing dynamic viscosity. The melt pool temperature mainly depends on the laser absorptivity of the metal rather than the Joule heating effect of the pulsed current.

By substituting Equation (9) into Equation (8), it can be rewritten as:

(10) $$\vec{F_{f}}=\frac{3\pi {\rho_{m}d}_{p}\mu }{4\rho_{g}}(\vec{u}-\vec{v})$$

According to the Stokes formula, relative flow velocity of the pore can be expressed as [28]:

(11) $$v=\frac{2{d_{p}}^{2}(\rho_{m}-\rho_{g})g}{9\mu }$$

Therefore, the distribution of pores in the laser cladding zone is mainly influenced by the current density and the pore volume. The electromagnetic repulsion generated by the pulsed current causes the internal gas to escape towards the cladding surface. The pores situated proximal to the periphery of the cladding zone, distal to the axis, are subjected to augmented electromagnetic repulsive forces, thus making the process of escaping from the surface of the cladding zone faster. In contrast, the pores around the center of the cladding zone experience less electromagnetic repulsion, preventing them from escaping in time to remain inside the clad zone, as shown in Figure 4b. In the process of internal pore escape, many of the tiny bubbles come together and merge into a new one to create a larger volume, which accelerates their escape as shown in Figure 4c. The large pulsed current also favors increased electromagnetic repulsion and molten pool temperatures, hastening the escape of the pores that were previously situated at the center of the cladding zone. The quantity of pores in the cladding zone undergoes a notable decrease, while the volume of the pores is concurrently amplified, as shown in Figure 4d.

### 3.2. Phase Analysis

**Figure 7:**
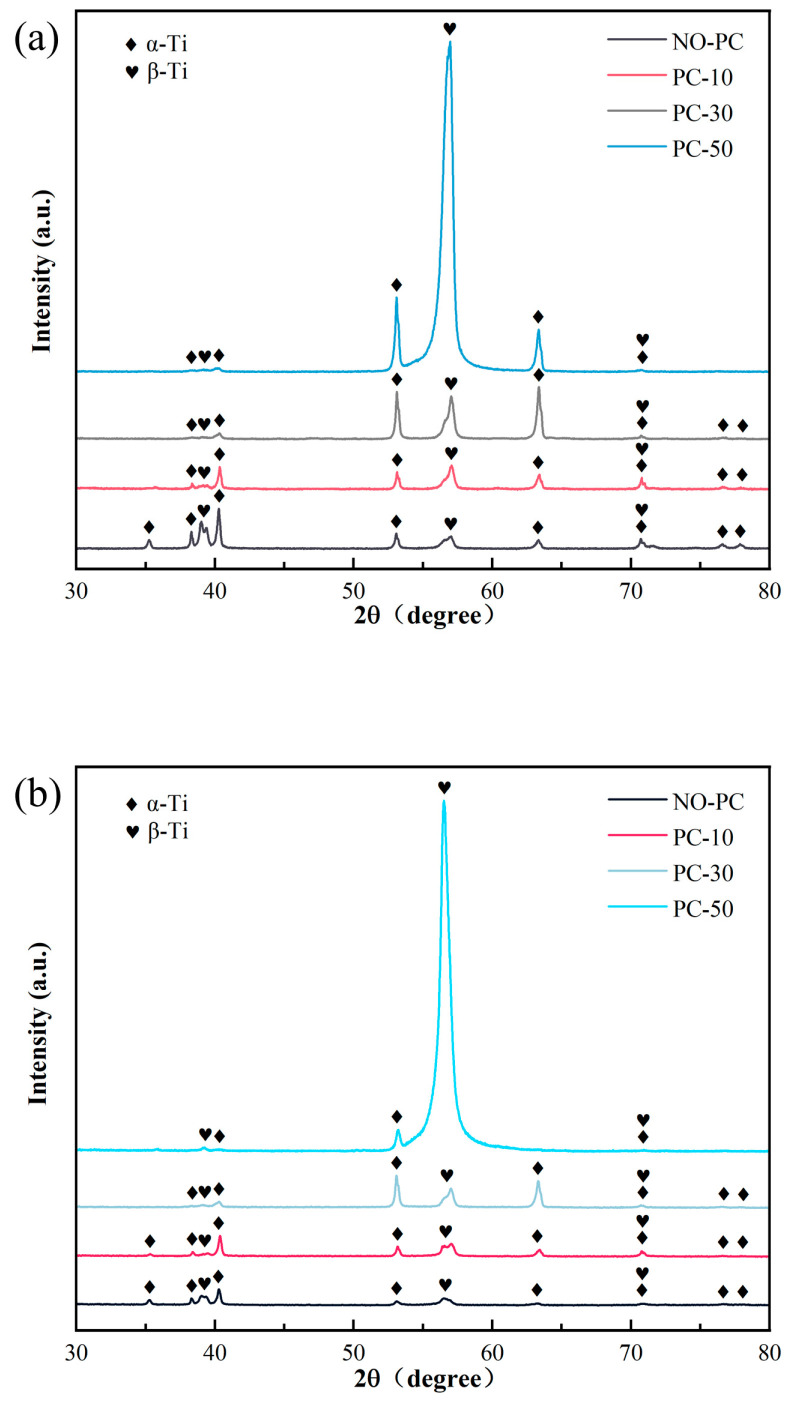
(**a**) The XRD diagram of SZ; (**b**) the XRD diagram of LCZ.

The XRD results in the LDZ and SZ of samples under different pulse current intensities are revealed in Figure 7. The samples mainly contained α and β phases. The pulsed current had no impact on the phase composition of either region. In the substrate zone (Figure 7a), the intensity of the highest diffraction peak experienced alterations, along with a decrease in some peak intensities of the Ti-α phase. This is mainly attributed to the reduction of temperature at the α-phase to β-phase transition caused by the pulsed-current-induced “electron wind” effect, promoting the phase transition from α-phase to β-phase [29,30]. Increasing the intensity of the pulsed current enhances this effect, leading to a greater proportion of the β-phase. In addition, the peak intensity of the α phase at 53°and 64° was increased by the enhancement of current density. An asymmetry in the peak shape of the current-assisted occurred compared to the no-current, indicating that the secondary α-phase had been generated. With the influence of an electric current, the β-phase tends to transform more easily into the secondary α-phase on cooling [31].

In the laser cladding zone (Figure 7b), the diffraction peak variation of the samples followed the same trend as that of the substrate zone samples. Nonetheless, the α-Ti diffraction peak intensity in the laser cladding zone was markedly reduced compared to the substrate zone, a phenomenon attributable to the higher temperature characteristics of the former. It is easier for the temperature to go beyond $T_{\beta }$ in this zone, instigating a transition from the primary α-phase to the β-phase.

### 3.3. Microstructure

#### 3.3.1. LCZ

**Figure 8:**
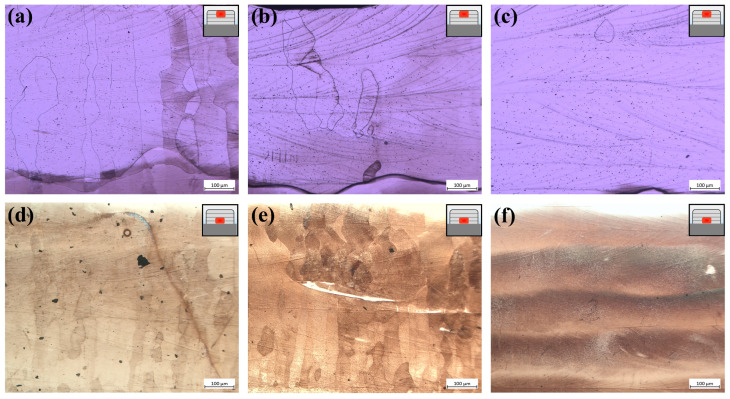
The microstructure of the LCZ cross-sections assisted by the pulsed current with different parameters; (**a**–**c**) the 3–4 cladding layer zone of the NO-PC, PC-10, and PC-50; (**d**–**f**) the 1–2 cladding layer zone of the NO-PC, PC-10, and PC-50.

The microscopic cross-sectional views from the LCZ of samples with different pulsed current parameters are shown in Figure 8. In the 3–4 cladding layer zone, the micromorphology is depicted in Figure 8a–c. During the no-pulsed current, an abundance of columnar β grains is evident, extending in the direction of cladding as illustrated in Figure 8a. Subsequent to the pulsed current, a limited presence of short columnar β grains is observable, with a notable absence of discernible grain boundaries in most regions, as demonstrated in Figure 8b. With an intensified pulse current, the grain boundaries remain indistinct in this zone, as shown in Figure 8c. The micromorphology for the 1–2 cladding layer zone is delineated in Figure 8d–f. In the absence of a current, the region exhibits a plethora of columnar grains, as highlighted in Figure 8d. After the pulsed current, some equiaxed grains are manifested in this zone, as seen in Figure 8e. With an escalating pulsed current, the grain morphology becomes indiscernible, supplanted by the silhouette of the cladding strip, presented in Figure 8f.

**Figure 9:**
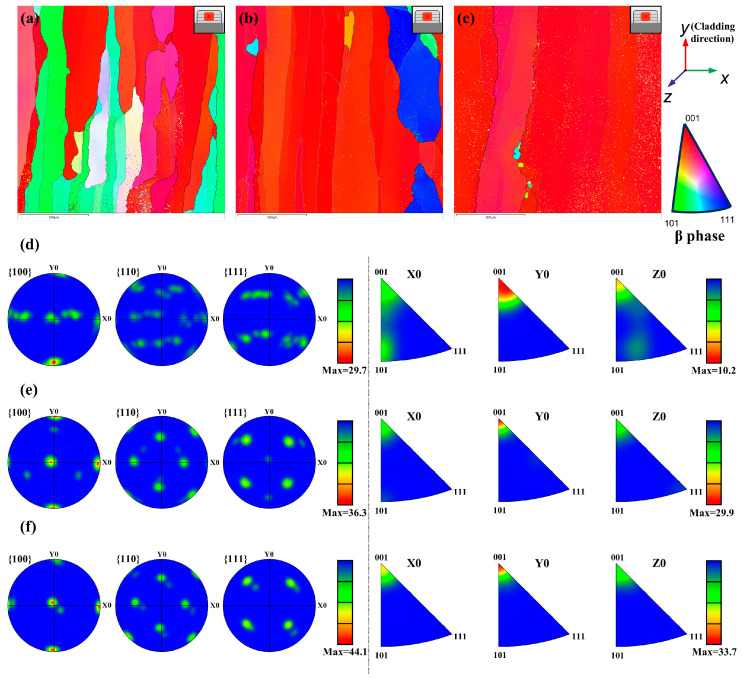
Grain orientations (IPF Z), pole figures, and IPFs of different samples in LCZ (**a**,**d**) NO-PC; (**b**,**e**) PC-10; (**c**,**f**) PC-50.

Figure 9a–c presents the inverse pole figure (IPF) map of the 3–4 cladding layer zone across varying samples. The micromorphology of cladding layers consisted of β grains interspersed with a minor fraction of the precipitated α phase. In the NO-PC sample, the texture predominantly exhibited a non-directional disposition. Conversely, the introduction of a pulsed current markedly altered the orientation characterization of the microstructure. When the current was 10 A, a clear preferred orientation was produced. The orientation of most β grains tended to be consistent, resulting in the size of β grains not being clearly determined. This is the reason why grain boundaries cannot be clearly seen in Figure 8. The manifestation of this phenomenon was accentuated by the pulsed current escalating to 50 A. To further analyze the texture variation, the pole figure and inverse pole figure (IPF) of the β phase were examined, as shown in Figure 9d–f.

**Figure 10:**
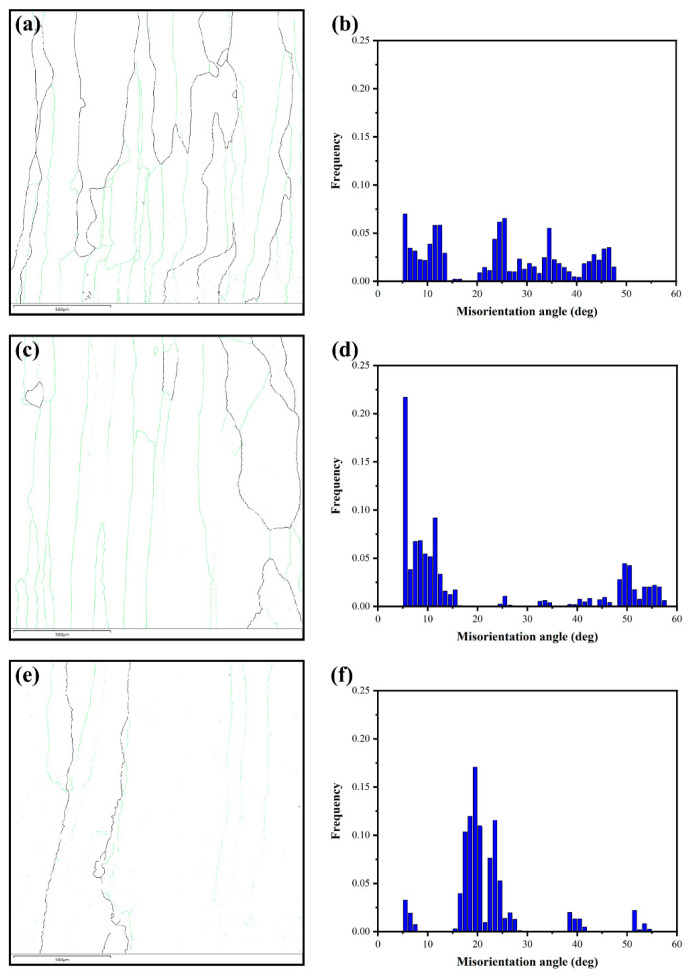
GB and misorientation angle maps of different samples in LCZ (**a**,**b**) NO-PC; (**c**,**d**) PC-10; (**e**,**f**) PC-50.

Without a pulsed current, the {100} < 001 > texture was formed along the Y0 direction, which was the cladding direction. However, the {100} < 001 > texture weakly exists. The texture distribution was relatively uniform, the orientations were along other directions, and the maximal texture index was 29.7. With the pulsed current assist, the texture along other directions became weakened, and the overall texture was stronger compared to Figure 9d. The maximum relative texture intensity was increased to 36.3, and the typical {100} < 001 > cube texture can be seen in Figure 9e. When the pulse current increased to 50A, the clearer {100} < 001 > cube texture was produced, and the maximum relative texture intensity was 44.9 (Figure 10f). The result indicates that the pulsed current promotes the growth of β grains with a <100>// cladding direction, and a higher current density induces more intense preferential growth.

Figure 10 shows the grain boundary distribution conditions of the HAZ of different samples, in which the colors represent different grain boundaries. The green solid lines represent low angle grain boundaries (LAGBs) with misorientation angles less than 15°. In contrast, the black solid lines represent high angle grain boundaries (HAGBs) with misorientation angles larger than 15°. The fractions of LAGBs, HAGBs, and grain boundary densities of β phases are shown in Table 4. Without a pulsed current, the fraction of HAGBs and the fraction of LAGBs of β grains was 44.0% and 56.0%, respectively. With the pulsed current assist, it can be seen that the fraction of HAGBs decreases and the fraction of LAGBs increases significantly, which was 21.9% and 78.1%, respectively. However, when the pulse current increases, the fraction of HAGBs with β grains in the PC-50 sample was elevated to 37.9%, while the fraction of LAGBs is reduced to 62.1% compared to the PC-10 sample. The previous study suggested that the pulsed current promotes a structural evolution in the material towards a state with an overall lower electric resistance [32]. This effect was also manifested in the altered state of grain boundaries.

For large angular grain boundaries (HAGBs), due to the strong discontinuity of the lattice at the grain boundaries, the grain boundary is a concentrated area of a large amount of lattice strain, and the grain boundary energy is high. A higher grain boundary energy sets up energy barriers for charge carriers’ transport between different grains, and the presence of the barriers strengthens the scattering to carriers, and thus reduces the charge carriers’ mobility in the material. For low angular grain boundaries (LAGBs), the discontinuity of the lattice at the grain boundary is weak, and the strain accumulated by the lattice discontinuity is mainly localized in the dislocation cores arranged “regularly” on the grain boundary and a very small region near them; the corresponding grain boundary energy is low. Lower grain boundary energy is not enough to form energy barriers that significantly affect the charge carriers’ transport, and it is therefore weak for charge carriers scattering, and has high mobility for the charge carriers in the material. Thus, when a pulse current was passed, the material itself will ensure the passage of majority charge carriers by decreasing the fraction of HAGBs and increasing the fraction of LAGBs. The mechanism of “selective” scattering of charge carriers by LAGBs will ensure electrical transport performance while reducing the thermal conductivity to achieve a balanced state with the electric-thermal transport performance (Figure 10d). The process manifests as the increase of the grain boundary angle, making the lattice mismatch between grains more serious. The density of “dislocation arrays” at the low angle grain boundaries also increases, which is conducive to the enhancement of the scattering of heat-carrying phonons while also increasing the grain boundary energy, resulting in a decrease in charge carriers’ mobility [33]. It can be seen that with the enhancement of the pulse current, the material itself reached an equilibrium state by decreasing the fraction of LAGBs and increasing the fraction of HAGBs. The grain boundary angles in the samples were concentrated around 21° (Figure 10f). At the same time, the GB-resistance also decreases with this grain boundary density. The grain boundary density of samples NO-PC, PC-10, and PC-50 were 12.70 × ${10^{-6}\cdot \mu m}^{-2}$, 8.79 × ${10^{-6}\cdot \mu m}^{-2}$, and 4.13 × ${10^{-6}\cdot \mu m}^{-2}$, respectively, as shown in Table 4. This further confirmed the previous results.

#### 3.3.2. SZ

**Figure 11:**
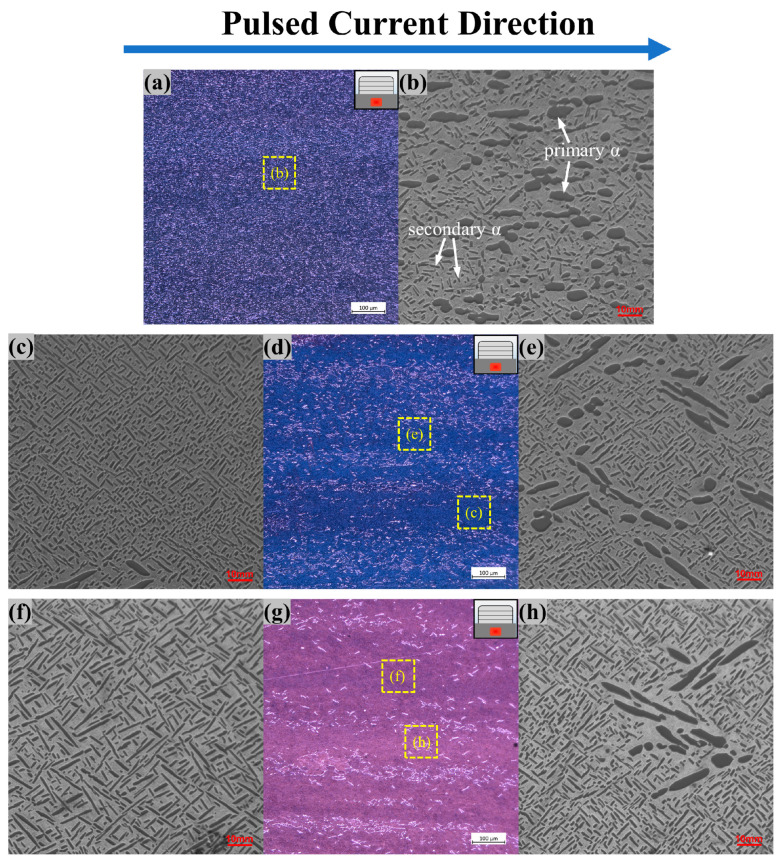
The microstructure of the SZ cross sections assisted by the pulsed current with different parameters (**a**,**b**) NO-PC; (**c**–**e**) PC-10; (**f**–**h**) PC-50.

The microscopic cross-sectional views from SZ of samples with different pulsed current parameters are shown in Figure 11. The NO-PC sample microstructure of the SZ was an α + β two-phase bimodal structure as shown in Figure 11a, and the primary α-phase was evenly distributed throughout the entire system. With the assistance of a pulsed current, the number of primary α-phases was significantly reduced, and tended to aggregate closely. Furthermore, the distribution of primary α-phases is aligned with the current direction, as shown in Figure 11d. As the pulsed current increased, this phenomenon became increasingly prominent, ultimately leading to the formation of a distinctive stripe-like configuration composed of the primary α-phase, as seen in Figure 11g. The magnified SEM images (Figure 11b,e,h) show that the primary α-phase, which was originally uniformly distributed, polymerized close to each other and changed shape from spherical to elongated. The pulsed current also promotes the development of the material to a state of lower overall resistance in the SZ. In the titanium alloy, the primary α-phase’s inherent low-resistance characteristic directs the current flow preferentially towards channels constituted by this phase [34]. Simultaneously, the close arrangement and elongated form of the primary α-phase within these channels optimize current distribution and further attenuate electrical resistance [35,36]. The region where less current passes is dominated by secondary α-phases, which increase significantly compared to the NO-PC sample, and have a basketweave structure (Figure 11b). The higher pulsed current can accelerate the atomic diffusion rate and promote a larger size of the secondary α-phase, resulting in changing its shape from a fine needle to a thin rod of a heightened aspect ratio (Figure 11f).

### 3.4. Microhardness

**Figure 12:**
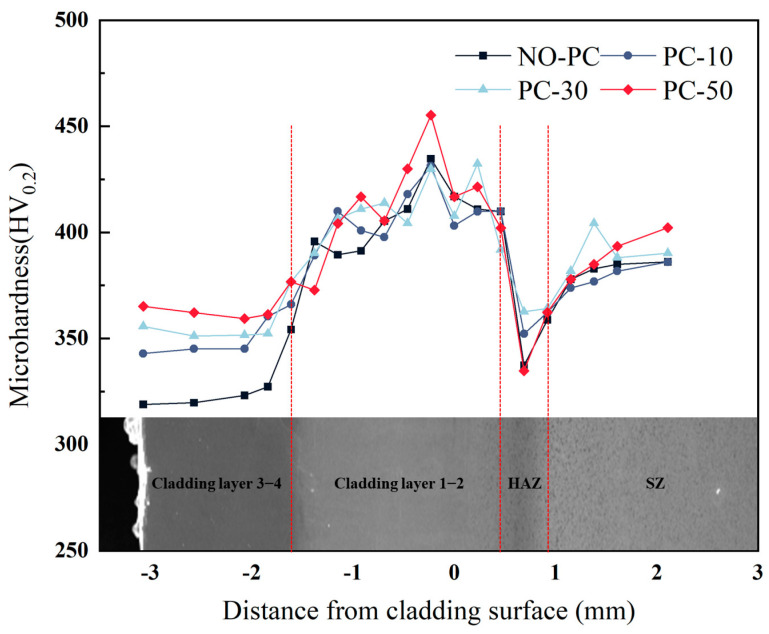
Microhardness profile of different samples from LDZ to SZ.

The microhardness profile from LDZ to SZ of different samples is shown in Figure 12. In the 3–4 cladding layer, there was a uniform distribution of hardness with depth. The microhardness showed an increasing effect with pulse current intensity. Specifically, the microhardness of the PC-50 sample was higher (365.1 ± 8 HV) than that of the NO-PC sample (318.9 ± 8 HV). As the depth increased, the 1–2 cladding layer’s microhardness showed a tendency to rise initially and subsequently decline. Mutual dilution of the substrate and cladding layer during laser cladding results in a considerable reduction in microhardness around the HAZ. In the substrate zone, the microhardness increase was not significantly affected by the pulsed current, the PC50 sample showed a mean hardness increase of 20 HV in comparison with the NO-PC sample. The microstructure indicates that the increase in microhardness of the PC-50 sample is a result of an increase in the amount of secondary α-phase. The larger size and more disordered arrangement of the secondary α phase in the PC-50 sample compared to the PC-30 sample enhances the dislocation restraining effect, resulting in a higher hardness. While the trend of the gradient microhardness remains unaltered by the pulsed current, the effect of the pulsed current results in an increase in the average microhardness.

### 3.5. Corrosion

**Figure 13:**
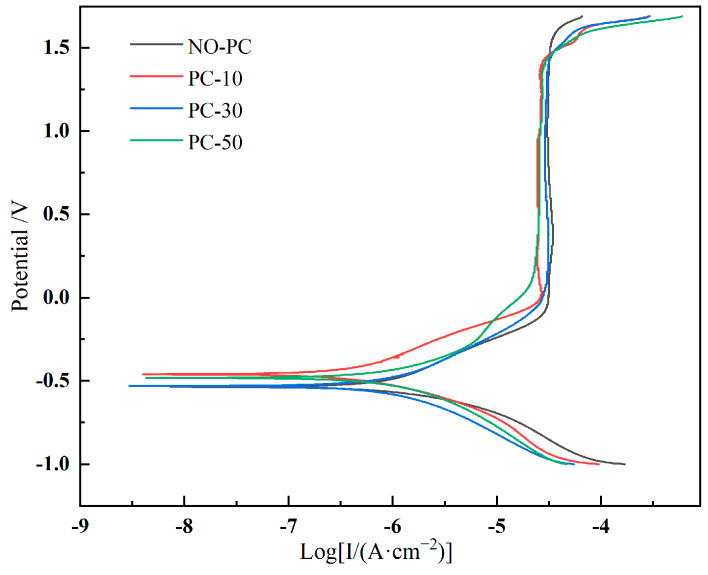
Polarization curve of different samples.

The dynamic potential polarization curve of different samples is shown in Figure 13. The corresponding electrochemical corrosion parameters are shown in Table 5. The NO-PC sample presented the highest corrosion current density ($I_{corr}$) of 6.31 × $10^{-7}$ A ${cm}^{2}$. The corrosion current density of the samples was reduced with the assistance of a pulsed current. However, the corrosion current density was noted to escalate with an upsurge in pulse current density. The PC-10 sample presented the lowest $I_{corr}$ of 2.81 × $10^{-7}$ A ${cm}^{2}$, and itself was the least susceptible to corrosion. All samples were passivated at 0 V. The highest passivation current density was observed for samples without pulsed current assistance. The sample without pulsed current assistance presented the highest passivation current density ($I_{pass}$) of 3.13 × $10^{-7}$ A ${cm}^{2}$. The passivation current density of the sample reduced with an increase in the pulsed current. The PC-50 sample presented the lowest $I_{pass}$ of 1.65 × $10^{-7}$ A ${cm}^{2}$, and it had better corrosion resistance after passivation. In summary, the corrosion resistance of the samples can be improved through the application of a pulsed current while cladding.

## 4. Conclusions

In this study, TC17 powders were cladded on a TC17 substrate assisted by a pulsed current. The cross-sectional morphology, porosity, grain orientation, grain boundary distribution condition, microstructure, and corrosion resistance at different pulsed current intensities were investigated. The technology is promising for the restoration of titanium alloy parts. The following conclusions can be drawn:

- Thanks to the influence of pulse currents on laser absorptivity, laser cladding assisted by a pulsed current can increase the dilution rate of the LDZ and reduce the width of the HAZ. Good metallurgical bonding could be obtained between the LDZ and SZ.
- Under the combined action of the Joule heating effect and the magnetic compression effect, the porosity and number of pores in the cladding area were reduced, and the ratio of equivalent pore size was increased.
- The pulsed current promotes the evolution of the material structure towards an overall lower resistance, including the preferential growth of β grains with <100>// cladding direction and a change of grain boundaries distribution in the LCZ, while there is the formation of a distinctive stripe-like configuration formed by the primary α-phase constituents in SZ.
- The average microhardness and the corrosion resistance of the sample were improved by the pulsed current. The improvements became obvious as the pulsed current increased.

## Figures and Tables

**Table 1:** Chemical composition of the TC17 alloy powder (wt%).

Element	Al	Sn	Zr	Mo	Cr	Fe	C	N	H	O	Ti
wt%	5.04	2.15	2.04	4.09	4.04	0.22	0.05	0.03	0.008	0.083	Bal.

**Table 2:** Pulsed current parameters in the experiments.

Sample	Peak Pulse Current (A)	Voltage (V)	Frequency (Hz)	Duty Cycle (%)
NPC	0	30	100	15
PC-10	10	30	100	15
PC-30	30	30	100	15
PC-50	50	30	100	15

**Table 3:** Geometrical dimensions of the relevant parameters of sample sections.

Sample	NPC	PC-10	PC-30	PC-50
W (mm)	9.509	9.377	9.699	8.975
H (mm)	2.781	2.913	3.093	3.013
D (mm)	0.647	0.684	0.736	0.728
d (mm)	0.254	0.251	0.265	0.238
η (%)	18.87	19.01	19.22	19.46

**Table 4:** Grain boundaries distribution and grain boundary density of β phases of LCZ under different samples.

Sample	LAGBs (%)	HAGBs (%)	Grain Boundary Density (${10^{-6}\cdot \mu m}^{-2}$)
NO-PC	56.0	44.0	12.70
PC-10	78.1	21.9	8.79
PC-50	62.1	37.9	4.13

**Table 5:** Electrochemical parameters based on potentiodynamic curves.

Sample	I_corr_( $\mu A/{cm}^{2}$)	I_pass_( $\mu A/{cm}^{2}$)
NPC	0.6314	0.3125
PC-10	0.2818	0.2623
PC-30	0.4687	0.2741
PC-50	0.5072	0.1651

## Data Availability

The data used to support the findings of this study are available from the corresponding author upon request.
